# High-Speed Impeller Design for the First Stage of a Hydrogen Compressor System

**DOI:** 10.3390/ma18174184

**Published:** 2025-09-05

**Authors:** Piotr Klimaszewski, Piotr Klonowicz, Łukasz Witanowski, Piotr Lampart

**Affiliations:** Institute of Fluid-Flow Machinery, Polish Academy of Sciences, Turbine Department, Fiszera 14, 80-231 Gdansk, Poland; piotr.klonowicz@imp.gda.pl (P.K.); lukasz.witanowski@imp.gda.pl (Ł.W.); piotr.lampart@imp.gda.pl (P.L.)

**Keywords:** hydrogen compressor, pipeline transport, material selection

## Abstract

Hydrogen compressors are key components of emerging hydrogen infrastructure. They are needed to meet the growing demand for hydrogen as an energy carrier. One of the challenges in their design is selecting a material and geometry for the impeller that ensures safe operation at high rotational speeds. This paper presents a numerical and structural analysis of a high-speed impeller designed for the first stage of a hydrogen compressor intended for pipeline transmission. The impeller geometry was developed using a 0D design algorithm and verified with CFD simulations. Stress and deformation were assessed using finite element method tools. The operating conditions considered were 28,356 rpm and a compression ratio of 1.25 at an isentropic efficiency of 75%. Four materials were analysed: aluminium 7075-T6, aluminium 2024 T851, stainless steel AISI 420, and titanium alloy Ti-6Al-2Sn-2Zr-2Mo. Equivalent stresses obtained from simulations were compared to the yield strengths of the materials. This study showed that aluminium 7075-T6 is the most suitable material due to its strength, machinability, and availability. It showed an equivalent stress of 398 MPa at a yield strength of 460–530 MPa. The results support the development of safe and efficient impellers for hydrogen compressors that can operate in future energy systems.

## 1. Introduction

Energy production represents a significant source of carbon dioxide (CO_2_) emissions on a global scale [1]. The transition to a low-carbon economy is a key objective of international climate policies, as outlined in agreements such as the Paris Agreement and in the European Union’s strategies for climate neutrality by 2050. In particular, green hydrogen—produced from renewable electricity—is expected to play a central role in decarbonizing sectors like heavy industry and long-distance transport. Hydrogen is increasingly recognized as a critical element in this transition. In consequence, over the past decade, considerable efforts have been made to promote and develop low-emission energy sources. As a result, the generation of energy from renewable sources has increased significantly on a global scale [2,3,4]. This is evidenced by reports from the Polish transmission system operator [5], which demonstrate that the installed capacity of wind power plants and other renewable energy sources has been growing rapidly for several years, as has the amount of electricity generated.

### 1.1. Hydrogen as a Green Energy Technology

One of the primary challenges associated with renewable energy sources is their variability. Until recently, power systems were primarily based on conventional power plants, which, due to their significant inertia, were unable to respond effectively to sudden fluctuations in electricity demand. At that time, pumped-storage power plants were employed to stabilise these fluctuations. In periods of energy overproduction, the pumping mechanism would be activated to transport water to an upper reservoir. In contrast, when energy consumption increased significantly, the descending water would drive hydro turbines, supplying electricity to the grid [6,7]. This system was designed to provide a balance between the morning “peaks” in demand and the afternoon “valleys”, which were caused by the technical limitations of conventional power plants.

In the current context, where the proportion of renewable energy is on the rise, it is also imperative to address the fluctuations in electricity generation. In the case of solar power plants, fluctuations can occur at multiple points throughout the day, with changes happening as frequently as every few minutes. The variability in generation is influenced by multiple factors, including cloud cover and the duration of sunlight, which varies with the seasons. In the case of wind-generated electricity, the challenges are not limited to short-term fluctuations in air speed. Additionally, periods of atmospheric calm or intense hurricanes, which may result in either a very low or an excessively high generation rate, must also be considered [8].

The introduction of renewable energy sources introduces an additional degree of instability to the power system. At the present time, the stability of the power supply is maintained during periods of low wind speeds or heavy cloud cover by conventional power plants that use coal, gas, or oil as fuel [9,10]. Pumped-storage power plants are used to compensate for short-term fluctuations in electricity production, which typically last several hours. Nevertheless, in instances where the aforementioned measures prove inadequate, the grid operator may implement supplementary mechanisms. Such measures include the disconnection of specific consumer groups, the import or export of emergency energy, and the activation of power plants that do not currently meet environmental standards or are in poor technical condition [11].

One of the most significant challenges currently facing the energy sector is the lack of economically efficient methods for storing large quantities of energy generated from renewable sources. In recent decades, numerous energy storage techniques have been developed, including both mechanical devices and chemical processes.

The authors in [12] present a study that examines the critical role of energy storage systems in addressing the inherent variability of renewable energy sources, such as solar and wind power. An in-depth analysis of electrical and thermal energy storage technologies is provided, focusing on their significance in enhancing grid stability and coordinating energy supply with demand.

Another article [13] introduces a multi-level isobaric adiabatic compressed air energy storage (MLIA-CAES) system designed to support standalone energy systems by balancing renewable energy availability and load demand. The system achieves over 83% round-trip and exergy efficiency under specified conditions, demonstrating both its operational flexibility and effectiveness.

Additionally, liquid air energy storage (LAES) is described by other authors [14]. According to their study, this technology offers a scalable solution for power management, with potential round-trip efficiencies ranging from 20 to 50% in decoupled systems and up to 50–90% in hybrid systems. Thermo-economic improvements include payback periods of approximately 20 years for standalone systems and 3–10 years for hybrid configurations, highlighting its potential for renewable energy integration.

Liu, H. et al. [15] detail a study on a Carnot battery system achieving 49.19% energy efficiency and 71.52% round-trip efficiency through advanced thermochemical and thermodynamic optimisations, providing insights into carbon-neutral energy solutions.

Finally, a review article [16] addresses the critical role of energy storage in sustainable development, particularly in developing regions. It highlights advancements in rechargeable batteries and emphasizes the impact of nanotechnology on improving battery efficiency, capacity, and lifespan, with a specific focus on the applications of alkaline and lead–acid batteries.

**Figure 1:**
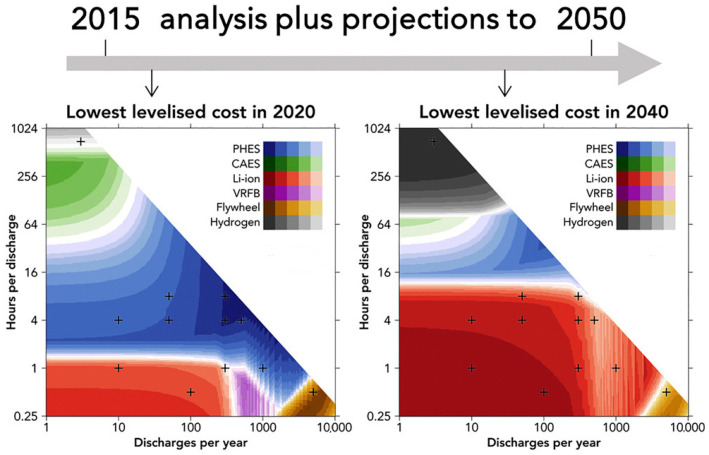
Cost of using individual energy sources depending on time and number of discharges per year [18].

In recent years, there has been a notable increase in the popularity of lithium-ion batteries. Despite the relatively high cost per unit of stored energy in comparison to mass, these batteries facilitate the stabilisation of short-term fluctuations in the power grid. One promising method for energy storage is the production of hydrogen through electrolysis, utilising additional electricity [17]. In this context, hydrogen functions as an energy carrier, exhibiting a capacity for storage analogous to that of hydrocarbons. The graphs in Figure 1 illustrate the potential for hydrogen-based technologies, as indicated by the projections regarding price changes for various types of energy storage. The authors in [18] predict that, as a result of the projected decline in costs, hydrogen will become an increasingly popular alternative to technologies based on air storage and pumped-storage power plants, potentially displacing these technologies from the market. Furthermore, the analyses indicate that hydrogen storage systems demonstrate robust performance during extended charging periods and with a limited number of charge and discharge cycles per year. The properties of hydrogen enable it to be liquefied and compressed, thus facilitating its storage in aboveground or underground tanks. During periods of surplus renewable energy production, hydrogen can be stored for extended periods. During periods of increased demand, it can be converted into electricity through fuel cells. Hydrogen is particularly promising as a solution to seasonal fluctuations in energy production, such as those observed in winter, when solar energy generation in Central Europe is low. Furthermore, hydrogen stored in dedicated tanks can be transported to areas with higher demand using vehicles or pipelines [19].

### 1.2. Hydrogen Transport

Pipeline transmission networks are currently regarded as the most cost-effective method of transporting materials over distances of up to 2500 km [20]. A substantial body of research is currently being conducted to examine the consequences of blending hydrogen with natural gas and the possible methods of its transportation. A substantial body of published literature suggests that existing natural gas pipelines can be modified to accommodate hydrogen transmission. Nevertheless, despite the advantages, there is also a considerable engineering challenge associated with guaranteeing the requisite level of safety and durability of the pipeline network [21,22].

A review of existing transmission networks has revealed limitations in the utilisation of current infrastructure, indicating a necessity for its expansion when the hydrogen concentration relative to methane exceeds a certain threshold. The safe level of hydrogen for transmission equipment and consumers is a topic of considerable discussion in the scientific community, with proposed levels ranging from 5 to 20% by volume of the gas [23,24].

In the case of hydrogen transportation via pipelines at higher concentrations, it is necessary to expand compression stations, given that hydrogen, due to its low molecular weight, necessitates the construction of more compression stations than natural gas. Furthermore, the utilisation of high concentrations of hydrogen may present challenges for gas consumers and potentially impact the durability of transmission system components. The advantage of blending hydrogen with natural gas is that it allows for direct transportation from the point of production to the consumer. This approach enables natural gas, a fossil fuel, to become a partially renewable fuel by incorporating “green” hydrogen. It is anticipated that there will be a notable increase in demand for pure hydrogen in the coming years. One article [25] suggests that modifying existing natural gas pipelines for hydrogen transport could cover more than 80% of the German network, reduce transmission costs by over 60%, and lower system-wide costs by 30% by 2030 compared to the construction of a new hydrogen pipeline infrastructure.

A compressor is a machine that is used for the transport of gases. Compressors can be classified according to a number of different criteria. One of the principal classifications is based on the design of the compressor, distinguishing between positive displacement compressors and dynamic compressors. In the category of dynamic machines, there are three main types: radial, axial, and centrifugal. The flow systems of these types of machines are subject to computational optimisation due to the variability of gas parameters and specific operating conditions [26,27]. The present article is concerned with centrifugal flow compressors operating at maximum load under nominal operating conditions. Due to the specific operating characteristics of this type of flow system, it is often utilised in the chemical industry and pipeline transportation [28,29].

The presented compressor impeller has been designed as a key component of a hydrogen compressor station, which forms part of the transmission network. Such designs must comply with specific requirements pertaining to the properties of the transported gas.

In the case of hydrogen compressors, the greatest challenge is the high peripheral speed of the impeller, which is a direct consequence of the low molecular weight of hydrogen. Consequently, it is essential to enhance the peripheral velocity, which can be accomplished by increasing the diameter of the impeller or its rotational speed. It is therefore essential to select an appropriate material for the hydrogen compressor.

The aim of this article is to provide a review of the materials that are most suitable for use in hydrogen compressors, with a view to selecting the optimal one. The topic of impeller shape has been the subject of considerable discussion amongst authors.

One illustrative example is the publication [30], in which the issues of the strength of axial turbine impellers were discussed. The authors presented an optimisation method with the objective of enhancing the rotor’s strength, with a particular focus on modifications to the computational grid in order to reduce the calculation time.

In the article by Zych et al. [31], the optimisation process of an impeller manufactured from Inconel 738 was discussed. The impeller was classified as highly loaded due to the elevated temperature in the operational environment (exceeding 600 °C) and the requisite rotational speed of approximately 96,000 rpm. The authors implemented a stress reduction strategy in the disk material through impeller shape modification, resulting in a stress reduction from 1288 MPa to 705 MPa.

The publication [32] concentrated on the structural strength analysis of a radial turbine impeller. In this instance, the optimisation process involved modifying the geometry and mass of the wheel. The authors were able to achieve an acceptable stress level and reduce the weight of the impeller in comparison to the reference design.

In [33], the issue of mixing hydrogen with methane was discussed, and a series of machine performance characteristics were prepared. The Mach number was posited as the threshold value. The results of the flow studies indicated that the rotational speed was significantly higher than that which standard materials (steel, aluminium, titanium) could withstand. The authors put forth the suggestion of utilising fibre-reinforced polymers (FRPs) as a means of fully realising the potential of the flow.

### 1.3. Objective and Novelty

The main objective of this study is to assess potential improvements in the design of impellers for high-speed hydrogen compressors by evaluating the suitability of various materials, including representative alloys from key material groups. The unique properties of hydrogen—particularly its low molecular weight and small atomic size—introduce significant challenges related to mechanical integrity, material selection, and economic feasibility. These challenges are further amplified at the high rotational speeds required for effective hydrogen compression, where impeller components are subjected to substantial mechanical stress [33]. While numerous studies have focused on the design and optimisation of impellers for neutral gases such as air or nitrogen, the specific requirements for hydrogen compression remain under-represented in the literature. The findings of this work are intended to contribute to the advancement of safe, efficient, and economically viable hydrogen infrastructure, supporting the broader adoption of hydrogen technologies within the energy sector.

## 2. Materials and Methods

This study presents the methodology used for the design and analysis of a hydrogen compressor impeller. The focus is on the first stage of a six-stage dynamic compressor that uses hydrogen as the working gas, with special attention paid to the selection and evaluation of suitable materials for the impeller. The applied methods include an original algorithm for geometry design, numerical simulations for validation, and strength calculations combined with material selection based on a material database.

### 2.1. Case Study—Hydrogen Impellers

The analysed impeller is the initial stage of the designed dynamic compressor. The compressor is a multi-stage design comprising six stages. An inter-stage gas cooler is employed to separate the first three stages from the subsequent three. In this instance, the working gas is hydrogen. The fundamental characteristics of the compressor were as follows: inlet pressure of 100 kPa, inlet temperature of 20 °C, and a mass flow rate of 0.5 kg/s.

**Figure 2:**
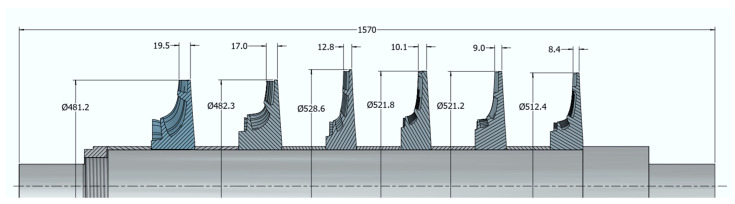
The main dimensions of hydrogen compressor impellers.

The flow geometry design was prepared using the 0D algorithm developed at the Institute of Fluid Flow Machinery, Polish Academy of Sciences, created in the Matlab R2021a environment [34]. A 0D meridional view of the compressor is presented in Figure 2, and the number of blades in the impellers, diffusers, and return channels of each stage is presented in Table 1.

The first stage was selected for detailed analysis because it experiences the highest loading conditions compared to the subsequent stages, which operate at lower compression ratios and are therefore less critical from a mechanical standpoint. In our design process, each stage was tailored individually to meet specific aerodynamic and structural requirements, ensuring that differences in blade geometry, rotational speed, and pressure rise per stage were properly addressed.

The first stage operates at a compression ratio of 1.25, with a rotational speed of 28,356 rpm, an impeller diameter of 481.2 mm, and an efficiency of 75%. These parameters result in the most demanding stress and deformation conditions across the entire compressor. The subsequent stages exhibit slightly lower compression ratios and power requirements, as presented in Table 2.

The 0D design was validated numerically using Ansys CFX 2021R1 software [35], with the RANS model, k-ω turbulence model, second-order upwind differencing scheme, and frozen rotor approach. The calculations were performed on refined meshes selected in the process of mesh independence studies, comprising approximately 1.8 million elements (for a compressor stage).

**Figure 3:**
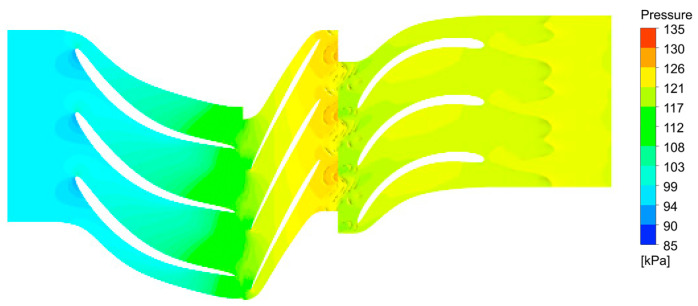
First compressor stage—pressure contours.

**Figure 4:**
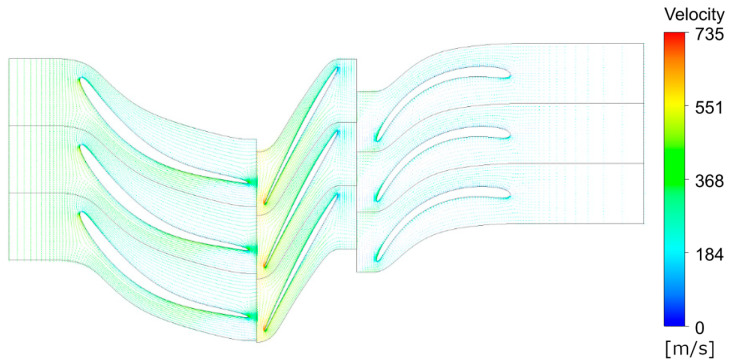
First compressor stage -velocity vectors.

Figure 3 shows the pressure contours along various segments of the first compressor stage. The results of the numerical simulations for the target configuration are presented in Figure 4, which illustrates the velocity variation across different sections of the stage in the form of vector fields. The efficiency found for CFD calculations of the first stage is equal to 79% as compared to 75% obtained from the 0D approach.

### 2.2. Strength Analysis—Choice of Mesh for the Analysis

A strength analysis of the impeller was conducted using FEM software—Ansys Mechanical 2023R2 [36]. The objective of this study was to identify areas that may be susceptible to deformation and subsequent rupture. Thermal stresses resulting from a slight temperature increase at the initial stage did not exert significant influence on the material behaviour; therefore, they were not taken into account in the analysis. The first stage of the strength analysis of the impeller was to perform a mesh quality assessment. For this purpose, five meshes with different numbers of elements were prepared (see Table 3), with mesh number 2 being the reference mesh. The number of nodes in this case was 766,121, while the number of elements was 516,695.

The quality of elements significantly decreased near the outer edge of the impeller and in areas where the element size is disproportionately large, mainly in the region between the blades. In the area of the blade edge, the quality of the elements is high, and no elements with a quality lower than 0.5 are visible.

**Figure 5:**
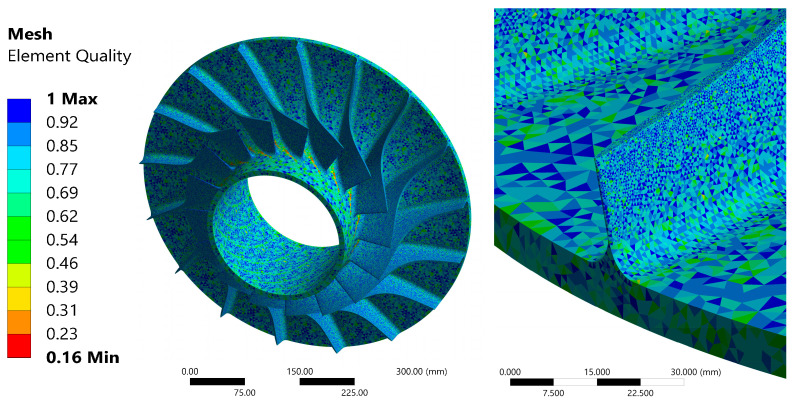
Computational mesh of the impeller (**left**), with augmentation at the compressor blade (**right**).

The precise configuration of the mesh elements within a single computational element is illustrated in Figure 5. The mesh is presented from two perspectives: a lateral view and a view of the blade inlet area. In order to maintain an appropriate mesh quality, the number of elements was increased in the regions of the blade edge and its lower side.

In preparing the stress analysis, it was necessary to verify the quality of the computational mesh. The utilisation of multiple levels of mesh refinement resulted in alterations to both the outcomes obtained and the time required for the calculations to be performed. The reference mesh comprised approximately 500,000 elements and 750,000 nodes and served as the basis for testing alternative mesh versions.

The mesh utilised in this study was evaluated for quality parameters (Table 4) through the utilisation of tools accessible within the Ansys R2023 R2 software, with the specific parameters defined in accordance with the instructions outlined in the software’s user manual [37].

**Figure 6:**
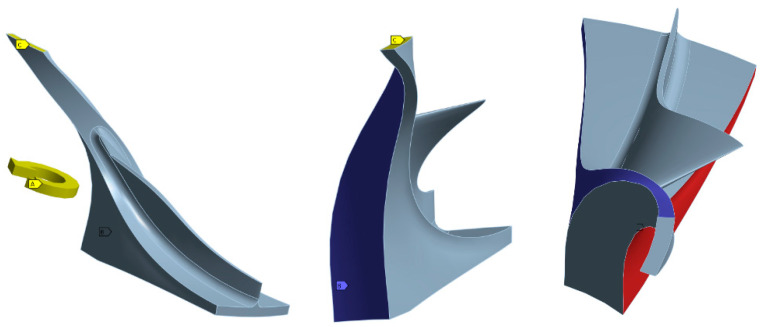
Boundary conditions—cylindrical support and displacement and cyclic region symmetry.

Boundary conditions for the calculations (Figure 6):

- Cylindrical support in the hole, with allowed rotation around the *z*-axis (axial direction) and fixed constraints in the radial and tangential directions. Blue colour (B symbol).
- Displacement of the back part of the disc in the axial direction is equal to 0 mm. Yellow colour (C symbol).
- Cyclic region symmetry. Red and blue colour.
- Direction of rotation (A symbol). Rotational speed: 28.356 rpm.

## 3. Results

This chapter presents the results of numerical calculations for various materials. The analysis begins with the reference case of an aluminium 7075 impeller. In the following section, the mechanical properties of alternative materials are evaluated. Based on the obtained results, seven alloys with the most favourable properties were selected. Furthermore, their respective advantages and limitations are discussed.

### 3.1. Reference Case

This study focused on the examination of materials that are commonly utilised within the industry for the manufacture of devices such as turbomachinery, engines, and compressors. The reference material selected was aluminium 7075 T6. This alloy, also designated as EN AW-7075 (EN AW-Al Zn5.5MgCu), is composed of the following primary alloying elements: aluminium (87.2–91.4%), copper (1.2–2%), magnesium (2.1–2.9%), and zinc (5.1–6.1%). The material is characterised by high mechanical strength; however, it is also subject to certain limitations, including low corrosion resistance and reduced performance at elevated temperatures. It is not advised to undertake welding of this alloy; however, its properties make it an appropriate material for machining. The mechanical properties of aluminium 7075 T6 are presented in tabular form in Table 5 (reference case).

**Figure 7:**
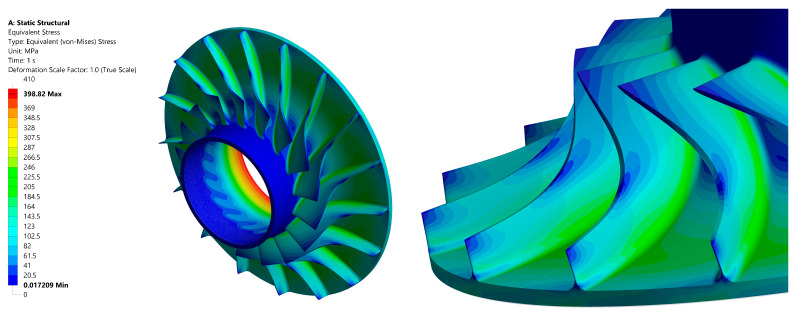
Stress distribution in the impeller: isometric view (**left**); at the compressor blades (**right**).

Figure 7 illustrates the distribution of reduced stress in the impeller. The highest levels of stress are observed at the blade root and in the central area of the rotor. Nevertheless, these stresses remain relatively low and do not exceed the maximum allowable limits for the material employed.

The results of the load distribution analysis suggest the potential for further optimisation of the rotor wheel’s shape. In areas proximate to the outer diameter, where forces are minimal, the potential benefits of incorporating specific notches (scallops) should be considered, as this could result in a reduction in mass and an enhancement of the overall structural efficiency of the component. The maximum calculated balanced load reached a value of approximately 400 MPa, which is well below the material’s strength limits. The yield strength of aluminium 7075 T6 is reported to range from 460 to 530 MPa, while its tensile strength is reported to be in the range of 530 to 580 MPa.

### 3.2. Selection of Alternative Materials

The objective of the numerical strength calculations was to identify fundamental parameters, such as the reduced stress value that occurs within the material. This value permitted the identification of boundary parameters that facilitate the subsequent phase of material selection, namely the analysis of alternative alloy materials.

The following methodology was deployed in the course of this analysis. Based on accumulated experience and established engineering practice, an initial reference material was selected, which is one of the most commonly used in such applications: this was the aluminium alloy 7075 T6.

In order to select the most appropriate construction material, the Ansys 2021 R1 package component, GRANTA SELECTOR [38], was used as the material database. The software contains a comprehensive database of materials, including metals and their alloys, along with a multitude of mechanical and physical properties. Additionally, it provides a set of filters that facilitate the selection of materials based on specific requirements.

**Figure 8:**
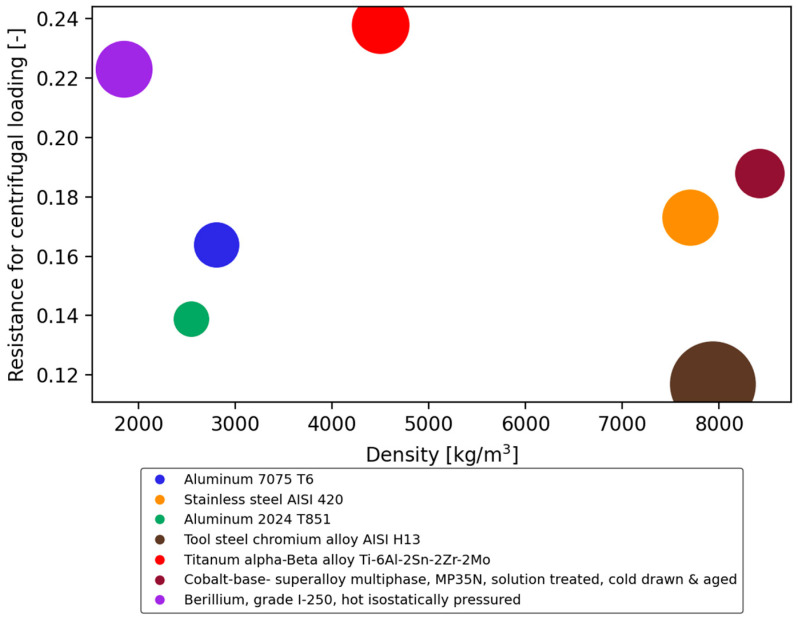
Resistance for centrifugal loads in relation to material density.

The subsequent phase of the process entailed the examination of potential alternative materials. In order to narrow down the selection, filters were introduced to the GRANTA software, which included factors such as the reduced stress value, yield strength, Young’s modulus, and operating temperature. A considerable number of potential materials were identified, classified into distinct categories and illustrated in the graphical representation presented in Figure 8.

The graph illustrates the results of the material selection process, as obtained from the Ansys 2021 R1 Granta software. The colours are used to represent different material groups:

- Red: Alloys that do not contain iron, such as cobalt, tungsten, and bronze alloys.
- Green: Iron-based alloys with carbon.
- Purple: Titanium, beryllium, and aluminium alloys.

The vertical axis defines resistance to centrifugal loading (RCL-dimensionless value), and the horizontal axis represents material density (ρ-in kg/m^3^).

One of the principal characteristics of an impeller is its resistance to centrifugal loading. It is defined as the ratio of yield strength to density (Equation (1)).

(1) $$\frac{\sigma }{\rho }=RCL$$

This parameter is therefore derived directly from the material properties, and the values used were obtained from the material database in ANSYS 2021 R1 GRANTA. The most desirable material was identified as one that combines relatively high resistance to centrifugal loading with low density. This choice ensures that the impeller can withstand the highest reduced stress values while maintaining structural efficiency.

The configuration of the graph in Figure 8 allows for the identification of the area where material groups that are suitable for the production of compressor impellers can be found. The most desirable material was defined as one that has relatively high resistance to centrifugal loading and low density. This defines the ability to withstand the highest reduced stress values. This is the region indicated in the upper left quadrant of the graph. The materials situated within this area include beryllium, aluminium alloys, and titanium alloys.

The materials initially considered in the preliminary analysis included metals, plastics, and composites. Based on the calculations performed for aluminium 7075 T6, the yield strength criterion was introduced, which significantly reduced the number of possible materials to approximately 4000 entries. This quantity was still too large for direct analysis, and thus another criterion was introduced, which was Young’s modulus (see Equation (2)):

(2) $$\frac{\sigma }{\varepsilon }=E$$

The Young’s modulus [39] is a characteristic property of each material and describes the relationship between deformation and stress within the material. Its unit is the Pascal [Pa = N/m^2^], which is defined as the force exerted per unit area.

The objective of introducing this coefficient was to eliminate materials that exhibit considerable deformation as a result of the forces acting on rotating components. The introduction of this criterion resulted in a notable reduction in the number of available materials, as the number of applicable plastics was found to be much lower than previously thought. At this stage, the number of materials that could be employed was in excess of 2000.

The next factor considered in the selection of materials was their ability to work in elevated temperatures. During the compression process, a notable increase in temperature is observed. Table 6 presents the temperature values for cases without inter-stage cooling and with cooling, obtained from MATLAB R2021a calculations.

In the initial phase of the design work, a system of inter-stage cooling was not planned; thus, a higher operating temperature was assumed for the final stage. The maximum safe operating temperature was set to 200 °C, which represented a significant limitation for many materials. The number of aluminium alloys and composites was reduced. Some of the most promising alloy types, including aluminium 7075 T6, were found to fail the criterion of safe operating temperature [40]. This alloy, in particular, is notable for its combination of high strength and low weight, making it a potentially advantageous material for the application in scope.

At this intermediate stage, the number of available material entries was reduced to approximately 1230. These included a variety of materials, including aluminium alloys, bronzes, and titanium alloys.

A preliminary analysis was made to check how the selected materials behave in contact with hydrogen. The phenomenon called hydrogen embrittlement (HE) means a reduction in plastic properties and the strength of metals because of the penetration of atomic hydrogen into the structure, which causes changes inside the material and leads to damage [41]. The analysis showed that all considered materials have some sensitivity to hydrogen, and in cases where this problem is important, the use of special protective coatings can be a possible solution.

For aluminium 7075-T6, contact with hydrogen causes a visible decrease in tensile strength and elongation. The fracture surfaces often show dimples, intergranular cracks, and striation-like patterns, which are typical for hydrogen embrittlement [42,43]. Aluminium alloys can suffer from hydrogen-assisted stress corrosion cracking, but in general, the negative effect is smaller than in titanium alloys, because hydrogen solubility and diffusivity in aluminium are quite low. Protective methods like anodized oxide layers or CrN coatings can reduce hydrogen penetration and improve resistance to embrittlement [44,45].

In the case of Ti-6Al-2Sn-2Zr-2Mo, the sensitivity to hydrogen is much higher. Titanium easily takes up hydrogen, which leads to hydride formation, microcracks, and embrittlement, especially during long-term loading or cyclic stresses. Both static and dynamic loads make crack initiation and growth faster in the presence of hydrogen. Although β-phase titanium alloys (with elements such as Mo and V) show better resistance than α-phase alloys, all titanium alloys can be damaged by hydrogen during long exposure [46,47].

To compare, titanium alloys are more sensitive to hydrogen embrittlement than aluminium alloys, because they can dissolve more hydrogen, form hydrides, and have phase-dependent microstructure effects. For this reason, in hydrogen-rich conditions, titanium parts need stronger protection, for example, special coatings and strict control of working conditions. If this is not possible, aluminium alloys can be a safer choice.

The compressor will have six compression stages, resulting in a temperature increase at each stage. A rise in temperature can be dangerous for many materials. For design reasons, the simplest solution is to use the same material to make all the impellers, which means that the thermal limits also apply to the first stage.

Ultimately, a decision was made to select a compressor design with inter-stage cooling, given the specific flow parameters. This resulted in the expansion of the material database. The database now comprised over 1000 entries, and it was therefore decided to select the most interesting or popular materials from each group for detailed analysis of their properties. The materials included in the study were as follows: beryllium grade I-250, aluminium 2024, aluminium 7075, stainless steel AISI 420, tool steel AISI H12, titanium Ti-6AL-2Sn-2Zr-2Mo, and cobalt superalloy MP35N. The chart presented illustrates the results generated using GRANTA software. The chart displays the properties of materials, with the areas illustrating resistance to centrifugal loading and density, as illustrated in Figure 8. The following section presents the results of numerical analyses for a selection of materials, as listed in Table 7. Three of the initially selected materials were not subjected to detailed numerical calculations. The properties of the final four materials are described in Table 8.

The following risks have been considered for the selected materials:

- Aluminium alloys (e.g., 7075-T6). Thermal softening at elevated temperatures can reduce fatigue strength.
- Titanium alloys. Hydrogen absorption can lead to embrittlement and reduced ductility over time.
- Martensitic stainless steel (AISI 420) is prone to hydrogen embrittlement under certain conditions. It also exhibits reduced toughness at low temperatures, which can increase susceptibility to brittle fracture under dynamic loading.
- Below are the risks for the materials excluded from this study:
- Beryllium grade I-250 is difficult to machine and has very limited availability, which significantly restricts its practical application [48]. Although beryllium is still used in certain highly demanding cases, its processing challenges limit widespread adoption. In terms of toxicity, beryllium exposure poses a significant health risk, primarily through inhalation. Airborne particles can accumulate in the body and cause serious respiratory and systemic effects. Soluble compounds may trigger allergic skin reactions, while insoluble forms can penetrate the skin. At elevated concentrations, exposure can lead to severe respiratory complications [49].
- Tool steel AISI H12. This steel is susceptible to hydrogen embrittlement in hydrogen-rich environments, leading to hydride formation, microcracking, and reduced fatigue strength. It is also prone to thermal cracking, loss of hardness during prolonged high-temperature exposure, machining difficulties, and corrosion in humid environments.
- Cobalt-based superalloy MP35N is a material that is relatively expensive and difficult to machine. The majority of the global cobalt supply originates from the Democratic Republic of Congo, accounting for a significant share of worldwide production, while Europe has virtually no substantial domestic sources. This situation, combined with concerns regarding the stability of supply chains as well as the social and environmental impacts associated with cobalt extraction in non-European regions, provides strong justification for excluding cobalt-based alloys from consideration in this study [50].

### 3.3. Aluminium 2024 T851

Another designation is EN AW-2024 (EN AW-AlCu4Mg1) [51]. This alloy consists of 90.8–94.7% aluminium, 3.8–4.9% copper, and 1.2–1.8% magnesium. The alloy’s properties, including its high fatigue strength and relatively low density, make it a suitable material for use in the aerospace and space industries, particularly in rotating components where fatigue resistance is important. It is one of the less expensive materials selected for analysis, and per unit volume it is the cheapest among those considered. However, it is important to note that this material presents certain disadvantages, such as being more difficult to machine than the reference material and requiring a welding process due to crack formation in the heat-affected zone [52]. The mechanical properties are presented in Table 9.

**Figure 9:**
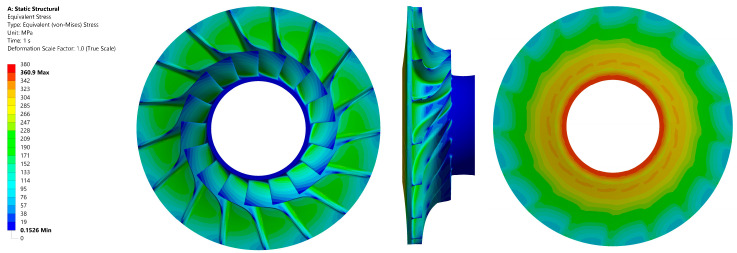
Stress values in the tested material (aluminium alloy 2024 T851) of the disc: front view (**left**); side view (**centre**); back view (**right**).

**Figure 10:**
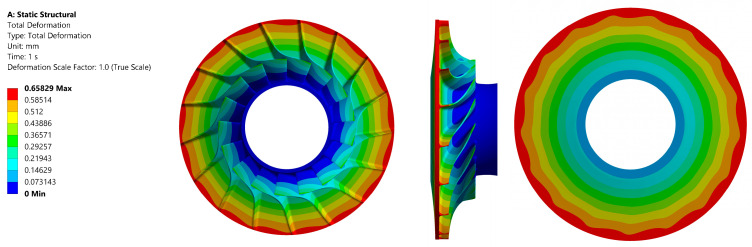
Total deformation in the tested material (aluminium alloy 2024 T851) of the disc: front view (**left**); side view (**centre**); back view (**right**).

The distribution of the reduced stress values in the material is presented in Figure 9. The calculations resulted in a maximum value of approximately 390 MPa. The area of maximum stress was identified as the inner diameter of the impeller. The total deformation view is presented in Figure 10.

### 3.4. Stainless Steel AISI 420

The material under discussion is a martensitic, magnetic stainless steel, which is commonly referred to by its other names: 1.4031, 1.4028, and 1.4024. Its composition includes 0.26–0.35% carbon, 12–14% chromium, and 82.6–87.7% iron. This alloy is extensively used in various industries due to its high hardness, which renders it suitable for use in moving machine components such as shafts and bearings. A significant application of this material is in the manufacture of knife blades. When priced per kilogram, it is one of the most affordable options in the set of materials under consideration.

**Figure 11:**
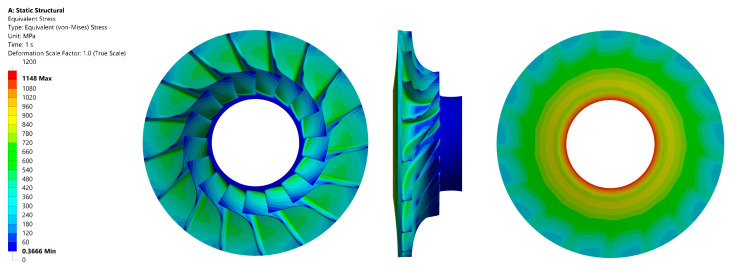
Stress values in the impeller with a disc material of AISI 420 alloy: front view (**left**); side view (**centre**); back view (**right**).

**Figure 12:**
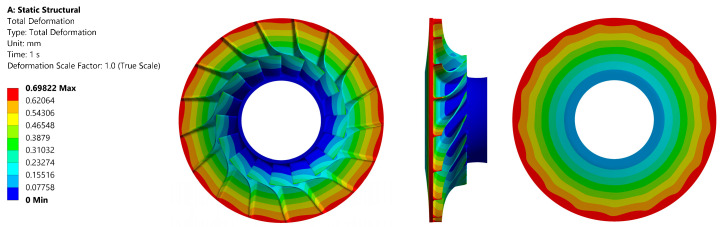
Total deformation in the impeller with a disc material of AISI 420 alloy: front view (**left**); side view (**centre**); back view (**right**).

In the analysed case, the maximum value of the reduced stress reached 1140 MPa, which was significantly higher compared to aluminium alloys. However, the material demonstrated compliance with the yield strength criterion, a phenomenon that can be explained by the superior strength of steel alloys in comparison to aluminium. The distribution of reduced stress in the material is illustrated in Figure 11, while the total deformation is presented in Figure 12. The fundamental mechanical properties of the alloy are outlined in Table 10.

### 3.5. Titanium Alloy

The alloy Ti-6Al-2Sn-2Zr-2Mo is a titanium-based alloy that is frequently used in the aerospace industry due to its high strength-to-weight ratio. This alloy demonstrates considerable resistance to cyclic loading, and its mechanical properties remain stable over a wide range of temperatures. The chemical composition of the alloy is 84–87.6% Ti, 1.75–2.25% Sn, 1.75–2.25% Zr, and 1.75–2.25% Mo. Although this alloy can be machined, welding presents greater challenges. Table 11 presents the selected mechanical properties of the alloy.

**Figure 13:**
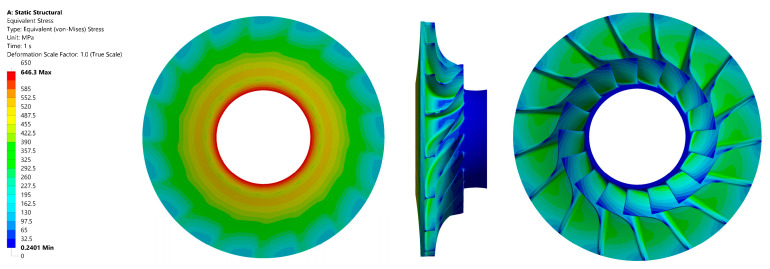
Stress values in the investigated alloy material Ti-6al-2sn-2zr-2Mo for the disc: front view (**left**); side view (**centre**); back view (**right**).

**Figure 14:**
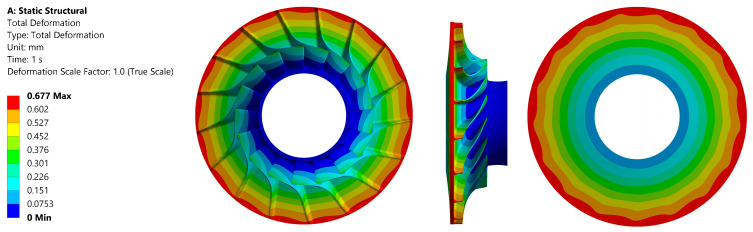
Total deformation in the investigated alloy material Ti-6al-2sn-2zr-2Mo for the disc: front view (**left**); side view (**centre**); back view (**right**).

The maximum value of the reduced stress observed in the material that was subjected to testing was 645 MPa. This value is comparable to that of aluminium alloys. It is evident that the material demonstrates considerably higher strength than the maximum loading value that was obtained. This finding offers a potential avenue for modifying the rotor design to achieve higher compression at the stage of the machine. However, this material is characterised by a significant drawback in terms of cost, which is several times higher than that of aluminium or steel alloys. The distribution of reduced stress in the material is illustrated in Figure 13, while Figure 14 presents the total deformation.

A further element of this study would be to conduct strength testing of the wheel on a dedicated test bench. Furthermore, the use of protective coatings on the working surfaces should be considered, as these would prevent degradation caused by hydrogen.

## 4. Discussion and Conclusions

The results of material selection for the design of a hydrogen high speed compressor impeller are presented in this paper. The impeller geometry, obtained through numerical simulations, was subjected to structural analysis. The selection of material was primarily based on the principles of equivalent stress and total deformation.

The choice of material has a significant impact on manufacturing constraints and overall lifecycle costs. For example, aluminium alloys like 7075-T6 offer good machinability and lower weight, which simplifies high-speed rotor fabrication and reduces energy consumption during operation. In contrast, materials such as titanium or beryllium alloys provide higher strength or stiffness but are more difficult to machine, often requiring specialized tools and processes, which increases production costs. Additive manufacturing can mitigate some geometrical constraints for complex impeller designs, but post-processing and surface finishing add to the total cost. Thus, selecting a material involves balancing mechanical performance, manufacturability, and economic feasibility to ensure industrial relevance.

The subject of the final analysis comprised seven materials, which represented different groups. A strength analysis was conducted on four of the materials. The remaining three materials were rejected on the basis of their inadequate machinability and high cost. These materials were as follows: beryllium grade I-250, tool steel AISI H12, and cobalt-based superalloy MP35N.

- The reference alloy identified for this study was aluminium 7075-T6, due to its high resistance to centrifugal loading. Material properties, including a density of 2800 kg/m^3^ and mechanical resistance values ranging from 0.164 to 0.189 (according to Ansys 2021 R1 Granta), were taken into account in the analysis. However, no significant performance advantage was demonstrated by aluminium 7075-T6 when compared to alternative materials.
- Titanium alloys, specifically Ti-6Al-2Sn-2Zr-2Mo, were subjected to rigorous scrutiny, with a particular emphasis on their performance under high-temperature conditions. The exclusion of inter-stage cooling in the initial design demonstrated the thermal advantages of titanium alloys over aluminium 7075. While both materials exhibit reduced yield strength at elevated temperatures, aluminium 7075 loses critical properties at approximately 200 °C. In contrast, titanium alloys retain mechanical stability, rendering them more suitable for such conditions. Nevertheless, its restricted availability and elevated machining costs posed challenges analogous to those encountered with beryllium.
- Aluminium 2024 T851 is a material that exhibits properties analogous to those of aluminium 7075. It is also the most economical material when compared with the reference material. The material exhibits certain drawbacks, including reduced machinability and a more complex welding process.

Following a rigorous process of evaluation, it was determined that aluminium 7075-T6 is the optimal material for the fabrication of the impeller. This decision was driven by its good mechanical properties but above all because of its wide availability, good machinability, and prior successful application in rotating machinery.

## Figures and Tables

**Table 1:** Number of blades in hydrogen compressor stages.

	Number of Blades		
	Impeller	Diffuser	Return Channel
**Stage I**	17	16	17
**Stage II**	15	16	15
**Stage III**	18	16	20
**Stage IV**	22	16	22
**Stage V**	22	16	22
**Stage VI**	22	16	22

**Table 2:** Compression ratio and power requirements.

Stage	Compression Ratio Π	PU [kW]
**I**	1.25	179
**II**	1.23	179
**III**	1.19	185
**IV**	1.23	210
**V**	1.21	210
**VI**	1.19	203

**Table 3:** Meshes considered in the quality study.

	Number of Nodes	Number of Elements
**1**	877.533	590.498
**2**	766.121	516.695
**3**	493.916	332.538
**4**	89.987	58.722
**5**	57.218	35.957

**Table 4:** Mesh quality parameters.

Parameter	Value
**Max aspect ratio**	14.714
**Min element quality**	0.139
**Min Jacobian ratio (corner ratio)**	0.084
**Min Jacobian ratio (guess points)**	0.425
**Max element edge length**	7.203 mm
**Min element edge length**	0.133 mm
**Max corner angle**	163.9°
**Max skewness**	0.972
**Min tet collapse**	0.131

**Table 5:** The main mechanical properties of aluminium 7075 T6.

Property	Value	Unit
**Young’s modulus**	69–76	GPa
**Ultimate tensile strength**	530–580	MPa
**Yield strength**	460–530	MPa
**Equivalent stress from numerical calculations**	398	MPa
**Vickers hardness**	152–168	[-]
**Fatigue strength at 10,000,000 cycles**	152–168	MPa

**Table 6:** The outlet temperatures for subsequent compressor stages.

Number of Stage	Temperature Without Cooling [°C]	Temperature with Cooling [°C]
**1**	45	45
**2**	70	70
**3**	95	95
**4**	129	74
**5**	163	103
**6**	195	131

**Table 7:** Materials obtained in the last phase of analysis.

No.	Material	Density	Resistance for Centrifugal Loading
**1**	Beryllium grade I-250, hot rolled	1850	0.223–0.263
**2**	Aluminium 2024 T851	2545	0.139–0.154
**3**	Aluminium 7075 T6	2800	0.164–0.189
**4**	Stainless steel AISI 420	7699	0.173–0.212
**5**	Tool steel AISI H12	7930–809	0.117–0.21
**6**	Titanium alloy α-β Ti-6Al-2Sn-2Zr-2Mo	4500	0.238–0.279
**7**	Cobalt-based superalloy, MP35N, solution heat treated, cold drawn, and aged	8415	0.188–0.218

**Table 8:** Main properties of finally selected materials.

Parameter	Material				Unit
**-**	Alu 7075 T6	Alu 2024 T6	Steel AISI 420	Ti Alloy	-
**Section**	3.1.	3.3	3.4	3.5	
**Young’s modulus**	69–76	72–75.7	195–205	113–124	GPa
**Ultimate tensile strength**	530–580	434–480	1550–1890	1240–1340	MPa
**Yield strength**	460–530	386–427	1330–1630	1070–1260	MPa
**Equivalent stress from numerical calculations**	398	392	1146	644	MPa
**Vickers hardness**	152–168	119–150	540–590	338–374	[-]
**Fatigue strength at 10,000,000 cycles**	152–168	120–154	581–669	514–523	MPa

**Table 9:** The main mechanical properties of aluminium 2024 T851.

Parameter	Value	Unit
**Young’s modulus**	72–75.7	GPa
**Ultimate tensile strength**	434–480	MPa
**Yield strength**	386–427	MPa
**Equivalent stress from numerical calculations**	392	MPa
**Vickers hardness**	119–150	[-]
**Fatigue strength at 10**,**000**,**000 cycles**	120–154	MPa

**Table 10:** The main mechanical properties of AISI 420.

Parameter	Value	Unit
**Young’s modulus**	195–205	GPa
**Ultimate tensile strength**	1550–1890	MPa
**Yield strength**	1330–1630	MPa
**Equivalent stress from numerical calculations**	1146	MPa
**Vickers hardness**	540–590	[-]
**Fatigue strength at 10**,**000**,**000 cycles**	581–669	MPa

**Table 11:** The main mechanical properties of Ti-6al-2sn-2zr-2Mo.

Property	Value	Unit
Young’s modulus	113–124	GPa
Ultimate tensile strength	1240–1340	MPa
Yield strength	1070–1260	MPa
Equivalent stress from numerical calculations	644	MPa
Vickers hardness	338–374	[-]
**Fatigue strength at 10**,**000**,**000 cycles**	514–523	MPa

## Data Availability

The original contributions presented in this study are included in the article material. Further inquiries can be directed to the corresponding author(s).

## Nomenclature and Abbreviations

The following symbols and abbreviations are used in this manuscript:

RCL	Resistance for centrifugal loading
RANS	Reynolds-averaged Navier–Stokes
FEM	Finite element method
CFD	Computational fluid dynamics
σ	Stress
ρ	Density
∅	Diameter
ε	Deformation
